# Ethical and methodological issues in qualitative studies involving people with severe and persistent mental illness such as schizophrenia and other psychotic conditions: a critical review

**DOI:** 10.1080/17482631.2017.1368323

**Published:** 2017-09-13

**Authors:** Ing-Marie Carlsson, Marjut Blomqvist, Henrika Jormfeldt

**Affiliations:** ^a^ School of Health and Welfare, Department of Health and Nursing, Halmstad University, Halmstad, Sweden

**Keywords:** Critical review, ethical issues, mental health, schizophrenia, trustworthiness

## Abstract

Undertaking research studies in the field of mental health is essential in mental health nursing. Qualitative research methodologies enable human experiences to become visible and recognize the importance of lived experiences. This paper argues that involving people with schizophrenia in research is critical to promote their health and well-being. The quality of qualitative research needs scrutinizing according to methodological issues such as trustworthiness and ethical standards that are a fundamental part of qualitative research and nursing curricula. The aim of this study was to critically review recent qualitative studies involving people with severe and persistent mental illness such as schizophrenia and other psychotic conditions, regarding descriptions of ethical and methodological issues in data collection and analysis. A search for relevant papers was conducted in three electronic databases, in December 2016. Fifteen qualitative interview studies were included and reviewed regarding methodological issues related to ethics, and data collection and analysis. The results revealed insufficient descriptions of methodology regarding ethical considerations and issues related to recruitment and sampling in qualitative interview studies with individuals with severe mental illness, putting trustworthiness at risk despite detailed descriptions of data analysis. Knowledge from the perspective of individuals with their own experience of mental illness is essential. Issues regarding sampling and trustworthiness in qualitative studies involving people with severe mental illness are vital to counteract the stigmatization of mental illness.

## Introduction

Knowledge of mental illness is primarily established by understanding of the perspective of individuals with experience of the phenomenon investigated (Mestdagh & Hansen, 2014). One of the largest differences between research involving the general population and research involving people with severe and persistent mental illness, such as schizophrenia and other psychotic conditions, is that stigmas about mental illness are widely endorsed by the general public and by mental health professionals (Corrigan, 2000; Hansson, Jormfeldt, Svedberg, & Svensson, 2011). This constitutes specific issues regarding research involving people with schizophrenia. People with severe and persistent mental illness are generally considered, completely or partly, unable to make autonomous decisions regarding their participation in research studies (Koivisto, Janhonen, Latvala, & Väisänen, 2001). Furthermore, parts of the data-collection process, such as the recruitment of participants, are often performed by healthcare staff who select participants and sometimes choose not to ask for participation, thereby working as a kind of gatekeeper who has the power to decide who should be given a voice in research and who should not (Allbutt & Masters, 2010). Gatekeeping in the research field of caring is frequently driven by the general assumption of vulnerability of patients, combined with an emphasis on the duty to protect patients. In addition, research is often perceived as a threat to patient well-being, while the benefits seem to be overlooked (Kars et al., 2016). Changing negative attitudes about mental illness will require changes in strategies to target effectiveness and efficacy issues in the carrying through of research studies and implementation of evidence-based interventions in mental health services (Corrigan, 2000; Hansson et al., 2011). A major concern in such research is that gatekeepers prevent the patients from making their own decisions regarding research participation, thereby overriding their autonomy (Kars et al., 2016).

Attaining trustworthiness in findings of qualitative studies is of great significance and the analysis approach used should assure trustworthiness in the data analysis regarding the perspectives of the individual, which is essential for developing nursing research and practice (Elo et al., 2014). Both inductive and deductive analysis processes represent three main phases, described as preparation, organizing and reporting the data (Elo et al., 2014), starting with selecting the units of meaning in the analysis. A unit of meaning in a qualitative analysis can consist of more than one sentence and contain several meanings, making the use of long units of meaning in the analysis process difficult and challenging (Graneheim & Lundman, 2004). However, too brief a meaning unit extracted from its context in the interview could result in fragmentation of the participant’s intended meaning (Elo et al., 2014). These issues become even more central when analysing data deriving from interviews with people who deliver different levels of incoherent speech or are taciturn at times despite being willing to participate and having important experiences to share. Detailed descriptions of the methodology used and the ethics considered are important to secure trustworthiness in qualitative studies to counteract stigmatization regarding mental illness.

The aim of the paper was to critically review recent qualitative studies involving people with severe and persistent mental illness such as schizophrenia and other psychotic conditions, regarding descriptions of ethical and methodological issues in data collection and data analysis.

## Method

### Identifying relevant studies

A search for relevant papers in was conducted in December 2016 in three electronic databases: CINAHL, PubMed and PsycINFO. To help with the search, the authors discussed search terms with an experienced librarian, who also conducted the final search. Our intention was to capture qualitative articles that used narratives or verbal data from adults diagnosed with schizophrenia or other psychotic conditions. To narrow and expand the search we used search tools such as medical subject headings (MESH), Boolean operators and truncation. In all databases, single and combined search terms included the keywords “schizophrenia”, “persistent”, “severe”, “focus group”, “interview” and “qualitative study”. Those publications defined as relevant were empirical peer-reviewed papers, published in English during 2012–2016. Inclusion criteria were articles in which people aged 18–65 years with schizophrenia participated in individual interviews. To ensure a focus on issues related to the qualitative interview dialogue between the participant and the researcher, studies excluded from this review were focus group interviews and interview studies with large sample sizes and quantitative data.

### Study selection

**Figure 1.**
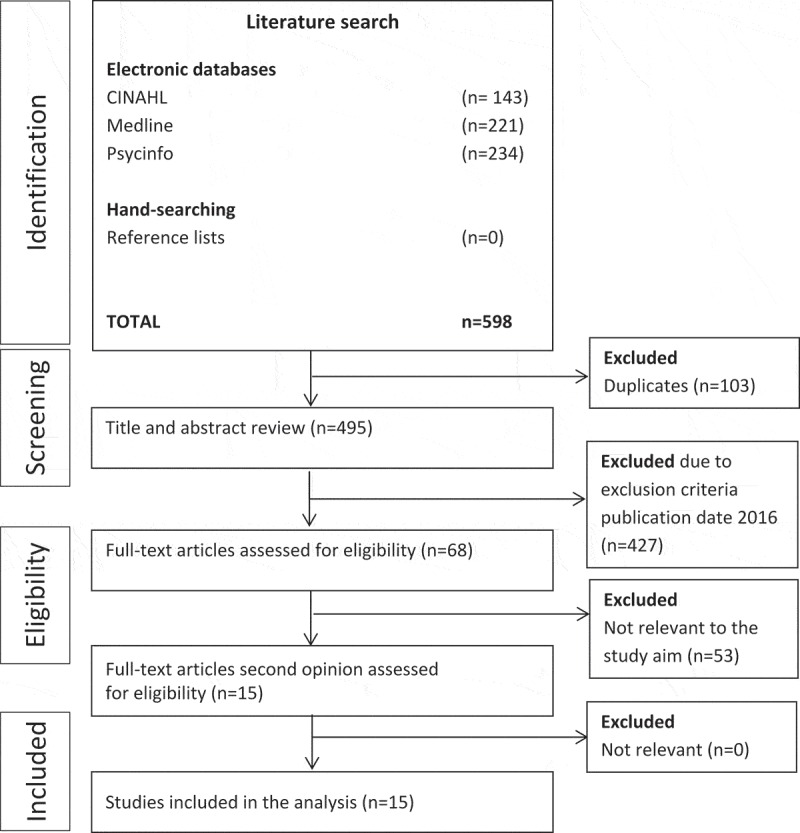
Flowchart of the literature search and selection.

The searches identified 598 articles, which were catalogued in EndNote®. Duplicates were then removed by automation supplemented with manual checking. Altogether, after scanning of the titles and abstracts, 495 articles remained as potentially relevant articles. At this point, we made a decision regarding the number of articles, to include only the most current articles, i.e., those published during 2016. The total number of eligible articles was 68, and the authors read the full text of all these. Articles that did not have relevance to the aim, for example, articles which interviewed relatives and healthcare professionals or focused on children and adolescents, were excluded. Finally, 15 articles were included in the present critical review (Figure 1). The authors read each method section and limitations section, if applicable, several times to become familiar with the content. Then, a summary tool was developed to extract information related to issues regarding ethics, data collection and data analysis (Table I). Included and reviewed articles are marked with an asterisk (*) in the reference list.

**Table 1.** Characteristics of the articles included in the critical review, including issues of data collection, data analysis and ethics.

Authors (publication year), country	Title	Aim	Method/setting	Sample/recruitment	Data analysis	Ethics	Methodological limitations and strengths
Barut, Dietrich, Zanoni, & Ridner (2016), USA	Sense of belonging and hope in the lives of persons with schizophrenia	To explore sense of belonging and hope in the lived experience of people with chronic schizophrenia spectrum disorders	Qualitative analysis; semi-structured interviews. A psychosis unit at the hospital	20 participants with chronic schizophrenia-spectrum disorder. Purposive sample. The physician gave feedback on the person’s ability to provide informed consent. Those who were determined to have capacity to consent were approached. The study participant received a 25-dollar gift card. One participant became notably anxious during the interview and ended the interview early	Line-by-line analysis using ATLAS. Two of the authors participated during the analysis	Institutional review board approval. Informed consent was signed	The authors discuss that the setting, a psychosis unit at the hospital, could have affected the results
Bjørkedal, Torsting, & Møller (2016), Denmark	Rewarding yet demanding: Client perspectives on enabling occupations during early stages of recovery from schizophrenia	To explore how people with schizophrenia experience an occupational therapy intervention designed to enable them to carry out meaningful occupation in the early phases of recovery	Feasibility study of an intervention; qualitative interviews. A large mental health centre	10 participants diagnosed with schizophrenia spectrum disorders. Convenience sample. Staff approached eligible candidates and then the researcher gave information	Thematic analysis. Three of the authors participated during the analysis in different steps	The Ethics Committee of the Capital Region stated that no approval was necessary. Informed consent was signed. The participants were informed that their participation would be described briefly in their medical records due to the intervention	The authors discuss interpretation bias. The recruitment strategy, sample size and that no record was written about dropouts are discussed. Only those who completed the entire intervention participated in the interview
Blixen et al. (2016), USA	Barriers to self-management of serious mental illness and diabetes	To asses perceived barriers to self-management among patients with both serious mental illness and diabetes to inform healthcare delivery practice	Phenomenological approach; semi-structured interviews. An urban safety-net care setting	20 participants with a diagnosis of severe mental illness, from a larger study. Convenience sample	Qualitative content analysis	Approved by the institutional review board of the participating institution. Informed consent was assigned	The authors mention that it is a small convenience sample
Burke, Wood, Clark, & Morrison (2016), USA	Experiences of stigma in psychosis: A qualitative analysis of service users’ perspectives	To explore experienced, perceived and internalized/self-stigma	Thematic analysis; semi-structured interviews. People with contact with mental health services were interviewed in their homes	12 participants diagnosed with schizophrenia, schizoaffective disorder or delusional disorder. Data were collected as part of another study with consecutive sampling	Thematic analysis	Unclear	The authors problematize that the sample was biased towards males and white ethnicity. They also highlight that the results of social exclusion and marginalization are most pertinent for people with severe mental illness
de Jager et al. (2016), Australia	Investigating the lived experience of recovery in people who hear voices	To investigate voice hearers’ lived experience of recovery	Narratives; semi-structured interviews	11 participants completed the Diagnostic Interview for Psychosis to ascertain that they met diagnostic criteria. Recruited from the Hearing Voices Network and Australian Schizophrenia Research Bank. One participant withdrew after being informed that the interview could cause distress. Reimbursement was offered for travel expenses and time spent taking part	Narrative analysis	Ethical approval was obtained from the University of Sydney Human Ethic Committee. Informed consent was assigned	The authors problematize the small sample size and self-selection. Participants were actively involved in the generation of narrative summaries and the narratives were member checked by participants
Jones et al. (2016), USA	“Did I push myself over the edge?”: Complications of agency in psychosis onset and development	To investigate the subjective experience of agency in the onset and early development of psychosis	Phenomenology; semi-structured interviews; included participatory techniques	19 participants diagnosed with schizophrenia spectrum and/or bipolar disorder with psychotic features. Recruited through flyers, the internet, community field sites, word of mouth and clinician referral	Grounded theory and qualitative phenomenology	Approved by the primacy investigator’s ethics board. Standard consent process	The authors claim that they did not intend to produce generalizable knowledge
Kidd et al. (2016), Canada	Locating community among people with schizophrenia living with a diverse urban environment	To provide an in-depth examination of the experiences, beliefs, behaviours and spaces that constitute community participation for a highly diverse group of people with schizophrenia	Longitudinal qualitative ethnographic design; semi-structured interviews were conducted three times over 10 months. Field notes were taken on foot or in transit in the community with participants	30 participants with schizophrenia or psychosis spectrum mental illness. Purposive sample. Participants were recruited by care providers at hospital, community services and boarding home	Ethnographic method	Ethical approval and issues unclear	The study was conducted in a single urban setting. The participants were engaged in discussions about the emerging categories and themes. Preliminary findings were also reviewed by an advisory group of people with lived mental illness
Landon, Shepherd, McGarry, Theadom, & Miller (2016), USA	When it’s quiet, it’s nice: Noise sensitivity in schizophrenia	To explore and document the experiences of noise sensitivity in people with schizophrenia	Semi-structured interview	7 participants diagnosed with schizophrenia or schizoaffective disorder. Recruited from mental health service providers and associated networks	Thematic analysis	Ethical approval and issues unclear. Participants were invited to bring a support person to the interview but all declined	The authors mention that the sample was small
Paul (2016), India	Responses to stigma-related stressors: A qualitative inquiry into the lives of people living with schizophrenia in India	To shed light on conditions and factors that either promote or hinder coping with stigma among people living with schizophrenia	Qualitative interpretive design considering multiple intersectionalities; face-to-face in-depth interviews, using an open-ended interview guide	20 participants diagnosed with schizophrenia and/or schizophrenia-related disorder. Purposive and snowball sampling strategies were used due to poor social visibility of people living with schizophrenia, and locating and recruiting them was challenging. Participants were recruited from private psychiatric clinics, mental health agencies and schizophrenia support groups	Thematic analysis	Ethical approval was received. Ethical principles were followed	Findings failed to capture experiences of those who received long-term institutionalized care. Interview notes were validated by the participants themselves
Rhodes, Parrett, & Mason (2016), UK	A qualitative study of refugees with psychotic symptoms	To examine the experiences of refugees with psychotic disorders	Interpretive phenomenological analysis	7 refugee asylum-seekers diagnosed in a psychiatric setting with psychotic symptoms. Clinicians suggested participants. 14 participants were suggested; 4 declined to participate and 3 did not fulfil the inclusion criteria	Interpretive phenomenological analysis	Ethical approval was granted by the local research committee of the relevant London NHS Mental Health Trust	The authors problematize the small sample size and that six were male
Saavedra, Lópes, Gonzáles, & Cubero (2016), Spain	Does employment promote recovery? Meanings from work experience in people diagnosed with serious mental illness	To explore the meaning attributed to work activity by subjects with serious mental illness, mainly schizophrenia	Phenomenological approach	21 participants with a diagnosed schizophrenia spectrum disorder. Participants were selected from a larger sample of a study	Social positioning analysis	Ethical approval was given and informed consent was assigned	No limitations are discussed
Singh, Jakhaia, Amonashvili, & Winch (2016), USA	“Finding a way out”: Case histories of mental health care-seeking and recovery among long-term internally displaced persons in Georgia	To present histories of care-seeking for and recovery from mental illness and psychosocial problems in the context of protracted internal displacement	Case study; semi-structured interviews	9 participants. Purposive selection from a larger sample. Recruited by clinicians	Grounded theory	Ethical approval is not declared. Ethics section is missing	No limitations are discussed. Credibility was used by member checks with a different subsample
Topor, Ljungkvist, & Strandberg (2016), Sweden	The costs of friendship: Severe mental illness poverty and social isolation	To explore the relationships between financial strain and social isolation	Grounded theory. 11 were living in their own apartment and 5 in group homes	16 participants. Participants were recruited from their municipalities’ specialist team	Thematic analysis	The study was approved by the Regional Ethics Committee in Lund	The authors problematize the sample size
van Langen, Beentjes, van Gaal, Nijhuis-van der Sanden, & Goossens (2016), Netherlands	How the illness management and recovery program enhanced recovery of persons with schizophrenia and other psychotic disorders: A qualitative study	To describe how the illness management and recovery programme enhanced recovery of people with schizophrenia and other psychotic disorders, from their own perspective	Descriptive phenomenological approach; one-to-one interviews	14 participants were asked and 8 participated. Recruited from one outpatient unit for people with a psychiatric diagnosis. Their mental health nurse performed recruitment	Colaizzi’s data analysis method, supported by MAXQDA software. At the end of each interview, findings were summarized and checked with the participant to ensure that the accuracy of the experience was grasped	The study was conducted in accordance with the Declaration of Helsinki. The authors consulted the Dutch Central Committee on Research Involving Human Subjects and concluded that ethical approval was not needed because the participants did not receive treatment and were not asked to behave in a particular way. Informed consent was assigned	Selection bias is highlighted since the trainers selected the potential participants
Waite et al. (2016), UK	The patient experience of sleep problems and their treatment in the context of current delusions and hallucinations	To explore patients’ accounts of sleep problems and associated psychological treatment	Phenomenological approach; semi-structured interviews	10 participants with psychosis were recruited from a randomized control study	Interpretive phenomenological analysis	Ethical approval was obtained from an NHS research ethics committee	The participants were a selected group in the context of a research trial

NHS, National Health Service.

## Results

The results presented reflect a critical review of the ethical and methodological issues described in the studies related to the ethics, data collection and analysis of data from interviews involving people diagnosed with severe and persistent mental illness such as schizophrenia or other psychotic conditions.

### Ethical issues

In most countries, the legal framework governs research activity with humans; in Sweden there is a specific law for ethical approval in research on humans (SFS 2003:460, 2003). However, there are differences between countries regarding whether ethical approval is needed or not, sometimes depending on whether a study is qualitative or quantitative. In the present critical review, most studies, but not all, had approval from research ethics committees. Some of the reviewed articles were part of a larger study and these articles did not clearly state if the actual study had ethical approval or not. The reason for this could be that the actual qualitative study was part of a larger research project that had gained ethical approval for the whole project (Burke, Wood, Zabel, Clark, & Morrison, 2016; Kidd et al., 2016; Singh, Jakhaia, Amonashvili, & Winch, 2016). Two of the reviewed studies, one from Denmark (Bjørkedal, Torsting, & Møller, 2016) and one from the Netherlands (van Langen, Beentjes, van Gaal, der Sanden, & Goossens, 2016), indicated that they had consulted their ethics boards, which had replied that no ethical approval was needed. Both of these studies, however, as well as most of the others included, were conducted in line with the principles of the Declaration of Helsinki (2013) and the key ethical principles of autonomy, confidentiality, protection, do no harm and informed consent. Notable, however, regarding informed consent is that in one study the authors clearly stated that it was the person’s physician who gave feedback to the researcher on the person’s ability to provide informed consent, before the participant was even approached (Barut, Dietrich, Zanoni, & Ridner, 2016).

Moreover, in one of the present articles the authors acknowledged that participation in the interview study was described briefly in the personal medical records. The participants were informed, and the reason given was that the interview study was part of a larger intervention study (Bjørkedal et al., 2016).

### Issues of data collection

All of the reviewed studies gathered data through face-to-face interviews and it was common for the research team to use semi-structured interviews. The sample size varied between seven and 30 participants. In most of the articles, the authors discuss or mention the implications of a small sample size as a limitation for the research results (Bjørkedal et al., 2016; Blixen et al., 2016; Burke et al., 2016; de Jager et al., 2016; Jones et al., 2016; Landon, Shepherd, McGarry, Theadom, & Miller, 2016; Rhodes, Parrett, & Mason, 2016; Topor, Ljungqvist, & Strandberg, 2016), whereas other authors do not discuss this issue, even if fewer than 10 participants were interviewed (Singh et al., 2016; van Langen et al., 2016).

The articles included in this review used different sampling methods such as purposive, convenience and snowball sampling. Given the complexity of sampling, Paul (2016) explicitly indicates that snowball sampling had to be used owing to difficulties in accessing or approaching participants. Other sampling strategies used to enhance recruitment included the study participant receiving a gift card or reimbursement for travel costs to participate (Barut et al., 2016; de Jager et al., 2016).

Several of the studies did not clearly outline the sampling procedure or how the participants were approached. In some cases, this was probably due to the included interview study being part of a larger study (Blixen et al., 2016; Saavedra, López, Gonzáles, & Cubero, 2016; Waite et al., 2016). Nevertheless, for most of the studies recruitment was performed by the healthcare staff or clinicians, who approached eligible candidates for the studies in their daily work (Barut et al., 2016; Bjørkedal et al., 2016; Jones et al., 2016; Kidd et al., 2016; Landon et al., 2016; Paul, 2016; Rhodes et al., 2016; van Langen et al., 2016).

Additional sources, besides healthcare staff, used to approach participants were different networks (de Jager et al., 2016), flyers, the internet, community field sites and word of mouth (Jones et al., 2016).

### Issues of data analysis

#### Using more than one author to aid understanding of the content in the material

The method sections contained descriptions stating that the findings were scrutinized by means of discussions between authors during and after the analysis process. This process needs to maintain the original meaning of the statements when they are condensed, abstracted and labelled at a more abstract level to contribute to structured knowledge in the studied field. One of the authors in the reviewed articles was usually responsible for the main analysis and the other authors served as additional evaluators in the analysis procedure (Bjørkedal et al., 2016; Kidd et al., 2016; Paul, 2016; Rhodes et al., 2016). Others went a step further, and included more than one author to independently read the transcripts and initial analysis (Barut et al., 2016; Blixen et al., 2016; Burke et al., 2016; Jones et al., 2016; Landon et al., 2016; Saavedra et al., 2016; Topor et al., 2016; van Langen et al., 2016; Waite et al., 2016).

#### Retaining the original meaning of statements through the analysis process

The findings reveal that the authors made efforts to understand the content of the interview data and to avoid misinterpretation of the interviewees’ statements. Besides the authors cross-checking and discussing the content during the analysis process, some of the included studies used member checks to ensure the accuracy of the experience and to make sure that the underlying meaning of all the statements was preserved when they were abstracted into categories and themes (de Jager et al., 2016; Kidd et al., 2016; Paul, 2016; Singh et al., 2016; van Langen et al., 2016). This was done, for example, by summarizing the findings at the end of the interview or by checking field notes with the participant (Kidd et al., 2016; Paul, 2016; van Langen et al., 2016). Participants could also review and discuss preliminary findings as well as member checks with others who were not involved in the study, such as an advisory group of people or a subsample with experience of mental illness (Kidd et al., 2016; Singh et al., 2016).

## Discussion

Synthesizing and critiquing research is of importance to evidence-based practice but also with regard to ethics when human research is performed (Fothergill & Lipp, 2014). Three crucial areas regarding methodology in qualitative interview studies with individuals with severe mental illness were critically reviewed, relating to ethics, data collection and data analysis. Regarding analysis of data, two themes were described: using previous experiences to understand the content in the material, and retaining the original meaning of statements through the analysis process, which indicates awareness about the importance of interpreting the participant’s responses in accordance with his or her intentions. Thus, the importance of obtaining in-depth knowledge from the perspective of individuals with experience of the investigated phenomenon, as mentioned by Mestdagh and Hansen (2014), is well described and taken care of in some of the studied articles, for example through descriptions of member checks and the use of an advisory group of people with experience of mental illness (Kidd et al., 2016; Singh et al., 2016). In addition, attaining and reporting trustworthiness in the findings can be assured by the detailed descriptions of the analysis process in qualitative studies involving individuals with schizophrenia and other psychotic conditions, as highlighted by Elo et al. (2014).

Nevertheless, descriptions of gatekeeping and how protective power could be used by healthcare staff to restrict rather than safeguard patients’ opportunities to obtain improved services (Witham, Beddow, & Haigh, 2015) are not evident in the findings. Hence, it is noteworthy that in nine of the 15 studies recruitment was performed by the healthcare staff or clinicians (Barut et al., 2016; Bjørkedal et al., 2016; Jones et al., 2016; Kidd et al., 2016; Landon et al., 2016; Paul, 2016; Rhodes et al., 2016; Singh et al., 2016; van Langen et al., 2016).

In one study, the authors stated that it was the participant’s physician who gave feedback to the researcher on the person’s ability to provide informed consent, before the participant was even approached (Barut et al., 2016), and in another study participation was described briefly in the personal medical records (Bjørkedal et al., 2016). It is remarkable that these issues of power relations related to participation in research studies are not underlined, as it is of great importance that patients are given the opportunity to identify their own needs. Given the above, it seems plausible to argue that the research society needs to reflect upon implicit authority that may be built into healthcare organizations and affects who will be invited to take part in research and who will not. The lack of recognition regarding recruitment in qualitative studies is noticeable as it is often more challenging than expected and more significant than generally acknowledged (Kristensen & Ravn, 2015). One consideration is the need for careful reporting regarding how the selection process is conducted in qualitative studies involving individuals with severe and persistent mental illness such as schizophrenia and other psychotic conditions. Regarding ethical standards, it is worth mentioning that two of the reviewed studies, one from Denmark (Bjørkedal, Torsting, & Møller, 2016) and one from the Netherlands (van Langen et al., 2016), stated that they had consulted their ethical boards and no ethical approval was needed. This is true for many judgements from ethical boards when dealing with qualitative studies. However, our standpoint is that research involving people and their mental health issues is of a sensitive nature and should be subjected to ethical approval even if it is an interview study.

Furthermore, descriptions of how stigma regarding mental illness on different levels is handled to secure an objective, fair and equal recruitment of participants are frequently absent. Likewise, discussions of trustworthiness of findings related to internal stigmatization and other issues leading to flaws in communication skills among participants are often missing, as are descriptions of strategies and actions undertaken to control these concerns. These issues raise questions about the need for pronounced ethics regarding sampling procedures and related trustworthiness in qualitative studies involving people with severe and persistent mental illness such as schizophrenia and other psychotic conditions. Further studies regarding sampling procedures are urgently required to ensure trustworthiness in research originating from interviews with individuals who are largely dependent on decisions made by healthcare staff to be able to participate in research studies. Studies are also needed on the preparation, organization and reporting phases in the analysis of collected data to ensure the trustworthiness of findings regarding interviews involving people with severe and persistent mental illness such as schizophrenia or other psychotic conditions.

### Methodological strengths and limitations

This study used systematic searches to identify relevant articles. The selection criterion of publication date was initially broad. However, during the process, we recognized the need to focus and made a decision to include the most current research in the field, i.e., articles published during 2016. This strategy limited the number of articles that could have been of interest, but enabled us to be more critical. A limitation in this review is that not all included articles could be judged sufficiently owing to a lack of information. This may have affected how we presented and critiqued an article. However, if ethical or methodological issues are lacking or vague, this must be highlighted as a limitation in research.

## Conclusion

Knowledge from the perspective of individuals with experience of mental illness is essential. Thus, research requires the involvement of people with severe mental illness who can best inform the study. The main contribution of this review is to raise awareness of the importance of describing the decisions made and actions undertaken to obtain a proper sample, and finding solutions to difficulties in communication related to mental illness as well as social vulnerability among the target group, owing to dependence of care as well as inequalities in power regarding decision-making. Reporting ethical considerations and issues regarding recruitment and sampling is necessary for trustworthiness in qualitative studies to address issues of appropriateness and adequacy. Qualitative studies involving people with severe and persistent mental illness such as schizophrenia is vital to counteract the stigmatization of mental illness and improve living conditions in the community for these people.

## Notes

*Reviewed articles included in the Results section.
